# Research on Edge Service Composition Method Based on BAS Algorithm

**DOI:** 10.1155/2021/9931689

**Published:** 2021-07-15

**Authors:** Zhoujie Du, Huaikou Miao

**Affiliations:** ^1^School of Computer Engineering and Science, Shanghai University, Shanghai 200444, China; ^2^Shanghai Key Laboratory of Computer Software Testing and Evaluating, Shanghai 201112, China

## Abstract

Edge services are transferred data processing, application running, and implementation of some functional services from cloud central server to network edge server to provide services. Combined edge service can effectively reduce task computation in the cloud, shorten transmission distance of processing data, quickly decompose task of service request, and select the optimal edge service combination to provide service for users. BAS is an efficient intelligent optimization algorithm, which can achieve efficient optimization and neither need to know the specific form of function nor need gradient information. This paper designs an edge service composition model based on edge computing and proposes a method about edge service composition by BAS optimization algorithm. Our proposed method has obvious advantages in service composition efficiency compared with service composition method based on PSO or WPA heuristic algorithm. Compared with cloud service composition method, our proposed method has advantages of shorter service response time, low cost, and high quality of user experience.

## 1. Introduction

In recent years, researchers and scholars have made some achievements in research of QoS optimized service composition methods. Each task has multiple services that meet user's functional requirement in QoS optimized service composition execution workflow model. How to generate optimized composite services by using evaluation conditions of QoS solution. Considering QoS multidimensional attribute characteristics value in service composition, at present, there are main research methods including integer programming [1–3], mixed-integer programming [4–6], heuristic search algorithm [7–15], intelligent algorithm [16–23], etc.

In 2004, IBM T. J. Watson Research Institute Zeng et al. [1, 2] proposed an AgFlow service composition middleware platform, which modeled QoS-aware service composition problem as an integer programming problem driven by workflow, and used CPLEX solver to generate the best QoS composition service solution under business flow. Then, Deng et al. [3] proposed a QoS constraint-driven service composition optimization method in mobile cloud computing application environment. Ardagna and Pernici [4, 5] modeled service composition problem based on workflow as a mixed-integer programming problem and transformed cycle structure into a sequential structure, which reduced the problem of low performance caused by cycle. When user's global QoS constraints cannot be satisfied, the second optimization can reduce probability of service composition failure. Then, Wang et al. proposed an effective optimal service composition method [6] by using mixed-integer programming method from perspective of user trust and preference characteristics. Berbner et al. [7] proposed a heuristic search method considering constraints from QoS-aware service composition driven by workflow model. They used backtracking algorithm to generate effective service composition solution and selected optimal solution of service composition through branch limit method. Considering user end-to-end QoS constraints [8, 9], as well as shortcomings of global optimization and local optimization, Alrifai et al. [10] proposed a comprehensive Web service composition method combined global optimization and local selection technology. It used mixed-integer programming method to search global QoS constrained optimal solution under local constraints by local selection generated local optimal Web service. Under predefined workflow model, Hwang et al. [11] proposed a reliability measurement selection method to generate composite service solution that meets requirement by using finite state machine to represent call process among services. In order to further improve the reliability of service, Haddad et al. [12] proposed a service composition algorithm of local optimal selection from two perspectives of service transaction attribute and QoS. Subsequently, some researchers put forward efficient QoS optimization service composition methods from the perspective of local optimization selection [13–15].

Considering multidimensional nonfunctional characteristic attribute of service QoS, some scholars modeled QoS-aware service composition problem based on workflow as a multiobjective optimization problem [16, 17], which uses intelligent algorithms to solve and generate optimal composite service. Wagner et al. [18] proposed a QoS service composition model based on multiobjective optimization problem, and it considered multiple possible workflow execution structure at the same time. Cremene et al. [19] analyzed and compared QoS-aware Web service composition problem of the most popular centralized multiobjective optimization algorithm in the moment. Yilmaz et al. [20] proposed an improved genetic algorithm for QoS-aware to realize dynamic service composition. Kim et al. [21] put forward a method to automatically realize service composition in IoT application environment, which is based on efficient resource allocation method of genetic algorithm. Hossain et al. [22] proposed a large-scale data-driven service composition method by using parallel clustering particle swarm optimization algorithm in mobile environment. Peng et al. [23] implemented an adaptive service composition method by using distribution estimation of constrained Boltzmann machine.

As a novel stochastic optimization algorithm, Beetle antenna search (BAS) algorithm [24] is proposed in 2017 by Jiang and Li, which has a more concise search strategy based on the foraging behavior of beetles. The beetle antenna search can achieve efficient optimization does not need to know the specific function form and gradient information. Compared with particle swarm optimization (PSO), BAS only needs one beetle, which can reduce computational burden greatly. It does not know where food is when beetle foraging, but it can find food based on concentration of the food smell. The beetle has two long antennas; if smell concentration detected by left antennas is higher than that on the right, then the beetle will fly to the left; otherwise, it will fly to the right. According to this simple principle, beetles can find food effectively. The smell of food is equivalent to a function. Each point in the three-dimensional space function has a different value. Two antennas of beetle can sense two points of odor value near itself. The purpose of beetle antenna search is to find the point (where the food is) with the largest odor value in global space. We can optimize service composition efficiently by imitating the behavior of beetle foraging.

In this paper, we propose an edge service composition model based on edge computing and propose an edge service composition method based on BAS optimization algorithm. Beetle antenna search (BAS) algorithm [24] is proposed in 2017 (Jiang and Li), which has a more concise search strategy based on the foraging behavior of beetles.

## 2. Edge Service Composition

### 2.1. Edge Service Model

Edge computing is a new large-scale computing processing model. It integrates network, computing, storage, and application to provide edge intelligence service nearby. It can satisfy the key requirements of industry digitalization in agile connection, real-time business, data optimization, application intelligence, and security and privacy protection [25–27]. Cognitive computing on the edge for healthcare service robots can be processed by the robot without frequent communications with data centers [28]. An Edge Traffic Flow Detection Scheme and model proposed in [29, 30] can take full advantage of the computing resources of the surrounding vehicles and greatly reduce the execution time of the computation tasks.

**Figure 1:**
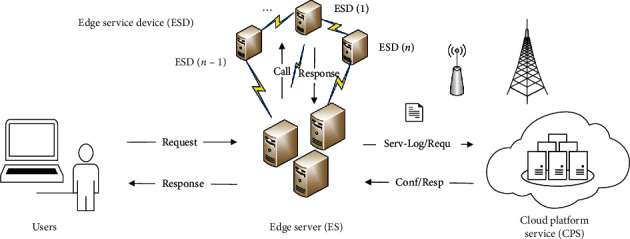
Edge service composition model.

Therefore, research on edge service composition method proposed in this paper based on edge computing mode uses superiority of edge computing and heuristic intelligent optimization algorithm under the environment of Internet. Edge service composition is generally divided into four main links: edge service request, edge computing, composite edge services, and feedback request processing result, reports the generated service logs to cloud service platform, reports confirmation, and so on. The most important difference between edge service composition and cloud service composition is that the edge service composition offloads work tasks (such as computing analysis) from cloud and loads it to edge server (that is, close to the end of service request). The advantage of edge service composition is that it can effectively reduce task computing scale in cloud, shorten transmission distance of service request, split service request quickly, and choose the best edge service composition to provide service for users, as well as improve quality of user experience greatly. Although the distance between edge and cloud is far, they are connected and dependent on each other. On the one hand, when a service request is sent out, the nearest edge server composites best service resource quickly to process request content and feeds back the execution result. On the other hand, edge server will report service status to be under the jurisdiction of cloud in the form of log, and cloud server will record service content, archive, and send confirmation feedback to edge server. Edge server will send a support request to cloud if composite edge service fails to meet a complex task request. The nearby cloud resources respond to request of edge server and accomplish request content together with edge server when cloud receive service request comes from edge server. In this paper, edge service composition model is proposed as shown in Figure 1.

Since QoS of service composition is evaluated from the perspective of nonfunctional attributes, so we use nonfunctional QoS attribute indicators to evaluate edge computing service composition in this paper. We evaluate and study QoS attributes such as service time (*T*), service cost (*C*), service availability (Av), service reliability (Rel), service reputation (Rep), and so on. The expression of QoS is shown in the following formula:

(1) $$\begin{array}{c} \text{QoS}=\left\{T\left(\text{ES}\right),C\left(\text{ES}\right),\text{Av}\left(\text{ES}\right),\text{Rel}\left(\text{ES}\right)\text{,Rep}\left(\text{ES}\right)\right\}. \end{array}$$

The attribute of QoS definition and quantitative expressions are as follows:

1. *T*refers to the total time expend from user submit service to accomplish service execution and return result. Generally, the shorter the service time is, the higher the service efficiency is.
2. *C*refers to all cost from user submit service request to accomplish service execution and return result.
3. Avrefers to the probability of service can be accessed successfully, that is, the ratio of the number of successful accesses edge service to total number of accesses, expressed as Av=*A*_*s*_/*A*_*n*_, and *A*_*n*_ is total number of accesses to the edge service over a period of time; *A*_*s*_ is the number of edge service successful responses.
4. Relrefers to the probability that edge server can provide service normally, that is to say, the proportion of service instance occupied time to total service working time, expressed as Rel=*T*_*r*_/*T*_*n*_, and *T*_*n*_ is the total running time of edge service;  *T*_*r*_ is normal operation time in this period of time.
5. Reprefers to measure trustworthiness of a service, that is, the evaluation made by users after using edge service, expressed as Re*p*=∑_*i*=1_^*n*^*R*_*i*_/*n*, where *R*_*i*_ is the evaluation of edge service made by *i*th user and *n* is counts for users evaluate edge service.

To build an QoS evaluation model of edge service, we can select a group of optimal service compositions from a large number of edge services by comparing QoS attribute values. However, because the significance of each QoS attribute is different, the representation method and quantization standard are also different. In order to eliminate influence of different dimensions, we need to normalize QoS.

The service time, service cost, and other attribute values of edge service QoS are expressed in the following normalization formula:

(2) $$\begin{array}{c} q_{i}^{-}=\frac{q_{\max}-q_{i}}{q_{\max}-q_{\min}}. \end{array}$$

The availability, reliability, reputation, and other attribute values of edge service QoS are expressed in the following normalization formula:

(3) $$\begin{array}{c} q_{i}^{+}=\frac{q_{i}-q_{\min}}{q_{\max}-q_{\min}}, \end{array}$$

where  *q*_max_ − *q*_min_ ≠ 0, *q*_*i*_^−^ or *q*_*i*_^+^ represent normalized value of edge service *i*th attribute, *q*_*i*_ represents *i*th QoS attribute value of edge service, *q*_max_ and *q*_min_ represent QoS attribute maximum and minimum in candidate set of edge service. It can be seen from the above formula that the normalized value of QoS attribute increases along with the increase of availability, reliability, and reputation and decreases along with the increase of service time and service cost. The QoS attribute values of edge service are all in the range of [0, 1] after they are normalized. We can unify processed QoS attribute values directly in this way.

### 2.2. Edge Service Composition Processes

**Definition 1 .**

(edge service (ES)). Edge service is provided by edge node server (close to service request) instead of central server in network deal with data or application operation for users. The edge service is formalized into a seven-tuple shown in the following formula:

(4) $$\begin{array}{c} \text{ES}=\left(\text{ID,CID,Fun,CN,}\frac{\text{Int}}{\text{Out}}\text{,Info,QoS}\right). \end{array}$$

where ID is identifier of edge service in edge service system, CID is the identifier of cloud service corresponding to the edge service, Fun is functional description of edge service, CN is the functional classification number of edge service, Int/Out represent input or output of service, Info is description of edge service attribute by provider, and QoS is quantitative evaluation of edge service quality.

**Definition 2 .**

(edge service candidate set (ESCS/ES)). It is a set of several edge services with same or similar functions that can complete a certain task independently; that is, edge services in the same set have same CN. Edge service candidate set is formalized as a triple shown in the following formula:

(5) $$\begin{array}{c} \frac{\text{ESCS}}{\text{ES}}=\left(\text{CN,Fun,Memb}\right), \end{array}$$

where CN represents classification number of edge service candidate set, Fun is functional description of all edge services in candidate set, and Memb is a sequence of all edge services IDs.

**Definition 3 .**

(composite edge service (CES)). CES is a logical service set composed of several sub-edge services with different functions, which can meet the user needs of handle complex tasks. The combined edge service can be expressed as a sequence shown in the following formula:

(6) $$\begin{array}{c} \text{CES}=\left({\text{ES}}_{1},{\text{ES}}_{2},\ldots ,{\text{ES}}_{n}\right), \end{array}$$

where ES_*i*_(*i* = 1,  2,  …,  *n*) is subservice of edge service combination and *n* is the number of subservices in edge service combination.

**Figure 2:**
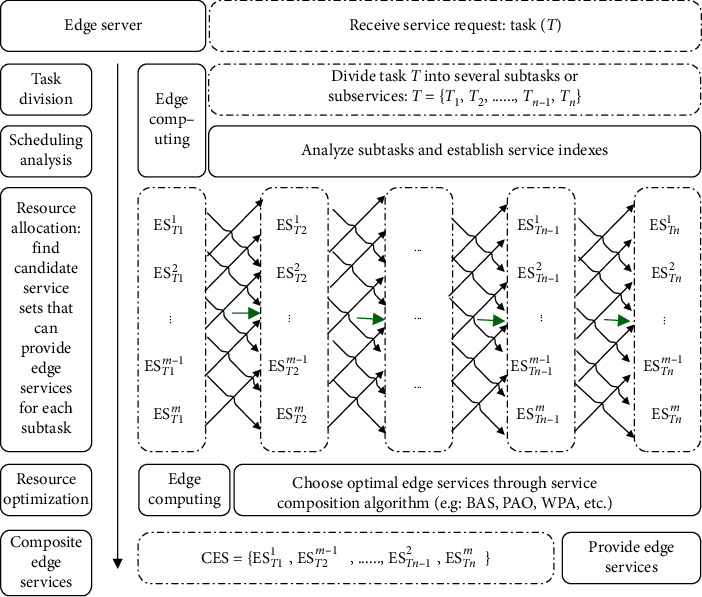
Edge service composition framework model.

Stable and efficient edge computing is the key to edge service complete various tasks, because single-function edge services cannot satisfy multifunctional needs of accomplished large-scale and complex tasks. It is necessary to combine multiple edge services according to certain logic to provide service to meet this requirement. Generally, we select a suitable edge service combination for a complex task through several main processes including task division, scheduling analysis, resource (or service) configuration, resource (or service) optimization, composite edge service, and general process as shown in Figure 2.

(1) Service division: the edge server divides service request into *n* subtasks that cannot be divided and can be executed by a single edge server after received service request. *T* represents the task of service request, and *T*_*i*_ represents subtask; then, *T*=∑_*i*=1_^*n*^*T*_*i*_, (*i* ∈ [1, *n*]).
(2) Scheduling analysis: edge server marks all edge service resources that can provide service for client requests through calculation and analysis, and edge service resources with the same or similar functions are marked as same type of resources.
(3) Resource (or service) configuration: ES_*T*_*i*__ represents edge services that can provide services for subtask (*T*_*i*_); there are *m* (*m* is an integer) edge services resources that can provide services to each subtask; then *T*_*i*_  corresponding candidate service set can be expressed as shown in the following formula:
  (7) $$\begin{array}{c} {\text{ES}}_{T_{i}}=\left\{{\text{ES}}_{T_{i}}^{1},{\text{ES}}_{T_{i}}^{2},\ldots ,{\text{ES}}_{T_{i}}^{m-1}{\text{,ES}}_{T_{i}}^{m}\right\}. \end{array}$$
If subtask *T*_*i*_ finally selects edge service ES_*T*_*i*__^*j*^  from candidate service set ES_*T*_*i*__ to provide services for it and enables the value of QoS (*T*_*i*_) to be optimized as well, then ES_*T*_*i*__=ES_*T*_*i*__^*j*^, and *i* ∈ [1, *m*].
(4) Resource (or service) optimization: we can use edge computing advantages to find a suitable edge service (ES_*T*_*i*__^*j*^ ) for each subtask (*T*_*i*_) through certain service optimization rules (such as beetle whisker algorithm, particle swarm algorithm, ant colony algorithm, wolf swarm algorithm, etc.) and then combine all selected services together and satisfy QoS (*T*) value optimal.
(5) Composite edge service: according to the results of resource optimization, choose a group of services composition to provide services for user requests and make QoS value optimal. CES represents composite edge service, and CES=∑_*i*=1_^*n*^ES_*T*_*i*__(*n* is the number of subservices). Let us suppose that ES_*T*_1__=ES_*T*_1__^1^, ES_*T*_2__=ES_*T*_2__^*m*−1^,…ES_*T*_*n*−1__=ES_*T*_*n*−1__^2^, and ES_*T*_*n*__=ES_*T*_*n*__^*m*^ after service selection optimization, and composite edge service set is expressed as follows:

(8) $$\begin{array}{c} \text{CES}=\left\{{\text{ES}}_{T_{1}},{\text{ES}}_{T_{2}},\ldots ,{\text{ES}}_{T_{n-1}},{\text{ES}}_{T_{n}}\right\}=\left\{{\text{ES}}_{T_{1}}^{1},{\text{ES}}_{T_{2}}^{m-1},\ldots ,{\text{ES}}_{T_{n-1}}^{2},{\text{ES}}_{T_{n}}^{m}\right\}, \end{array}$$

where *n* is the number of subtasks and *m* is the number of candidate services for each subtask.

### 2.3. The Formal Model of Service Composition

The QoS attribute value of composite edge service is not only related to QoS attribute value of single edge service but also related to structure between edge services. There are four basic structures: sequential structure, selective structure, parallel structure, and cycle structure in edge service composition. The QoS attribute aggregation function models corresponding to four basic structures are shown in Table 1.

In summary, we assume that each service composition has *n* unique edge services and has time, cost, availability, reliability, and credibility five QoS attributes, and any edge service composition path is *P*; then, the QoS expression is as follows:

(9) $$\begin{array}{c} \text{QoSP}=\left\{T\left(P\right),C\left(P\right),\text{Av}\left(P\right),\text{Rel}\left(P\right),\text{Rep}\left(P\right)\right\}. \end{array}$$

We can derive formula (10) by using formula (9), which is expressed as follows:

(10) $$\begin{array}{c} \text{QoSP}=F_{\text{Seq}}\left(T,C,\text{Av},\text{Rel},\text{Rep}\right)+F_{\text{Sel}}\left(T,C,\text{Av},\text{Rel},\text{Rep}\right)+F_{\text{Par}}\left(T,C,\text{Av},\text{Rel},\text{Rep}\right)+F_{\text{Cyc}}\left(T,C,\text{Av,Rel},\text{Rep}\right). \end{array}$$

The values of *F*_Seq_, *F*_Sel_, *F*_Par_, and *F*_Cyc_ depend on the structure of actual execution path of edge service, and *F*_Seq_+*F*_Sel_+*F*_Par_+*F*_Cyc_=1. We can derive formula (11) by using formula (9) and formula (10), which is expressed as follows:

(11) $$\begin{array}{c} \text{QoSP}=\left\{T_{\text{Seq}}\left(P\right)+C_{\text{Seq}}\left(P\right)+{\text{Av}}_{\text{Seq}}\left(P\right)+{\text{Rel}}_{\text{Seq}}\left(P\right)+{\text{Rep}}_{\text{Seq}}\left(P\right)\right\}\\ +\left\{T_{\text{Sel}}\left(P\right)+C_{\text{Sel}}\left(P\right)+{\text{Av}}_{\text{Sel}}\left(P\right)+{\text{Rel}}_{\text{Sel}}\left(P\right)+{\text{Rep}}_{\text{Sel}}\left(P\right)\right\}\\ +\left\{T_{\text{Par}}\left(P\right)+C_{\text{Par}}\left(P\right)+{\text{Av}}_{\text{Par}}\left(P\right)+{\text{Rel}}_{\text{Par}}\left(P\right)+{\text{Rep}}_{\text{Par}}\left(P\right)\right\}\\ +\left\{T_{\text{Cyc}}\left(P\right)+C_{\text{Cyc}}\left(P\right)+{\text{Av}}_{\text{Cyc}}\left(P\right)+{\text{Rel}}_{\text{Cyc}}\left(P\right)+{\text{Rep}}_{\text{Cyc}}\left(P\right)\right\}. \end{array}$$

We can derive formula (12) by using formulas (9)–(11), which is expressed as follows:

(12) $$\begin{array}{c} \left\{\begin{array}{c} T\left(P\right)=T_{\text{Seq}}\left(P\right)+T_{\text{Sel}}\left(P\right)+T_{\text{Par}}\left(P\right)+T_{\text{Cyc}}\left(P\right),\\ C\left(P\right)=C_{\text{Seq}}\left(P\right)+C_{\text{Sel}}\left(P\right)+C_{\text{Par}}\left(P\right)+C_{\text{Cyc}}\left(P\right),\\ \text{Av}\left(P\right)={\text{Av}}_{\text{Seq}}\left(P\right)+{\text{Av}}_{\text{Sel}}\left(P\right)+\text{Av}T_{\text{Par}}\left(P\right)+{\text{Av}}_{\text{Cyc}}\left(P\right),\\ \text{Rel}\left(P\right)={\text{Rel}}_{\text{Seq}}\left(P\right)+{\text{Rel}}_{\text{Sel}}\left(P\right)+{\text{Rel}}_{\text{Par}}\left(P\right)+{\text{Rel}}_{\text{Cyc}}\left(P\right),\\ \text{Rep}\left(P\right)={\text{Rep}}_{\text{Seq}}\left(P\right)+{\text{Rep}}_{\text{Sel}}\left(P\right)+{\text{Rep}}_{\text{Par}}\left(P\right)+{\text{Rep}}_{\text{Cyc}}\left(P\right). \end{array}\right. \end{array}$$

Since the parallel, selection, and cycle modes can be converted to sequence mode by using related technologies, we only discuss serial workflow mode in this paper. The goal of service composition is to minimize time and cost and to maximize availability, reliability, and reputation, so the expression of edge service composition QoS model is shown in the following formula:

(13) $$\begin{array}{c} \left\{\begin{array}{c} \text{QoS}\left(P\right)=\text{Min}\left(\varphi_{1}T\left(P\right)+\varphi_{2}C\left(P\right)+\frac{\varphi_{3}}{\text{Av}\left(P\right)}+\frac{\varphi_{4}}{\text{Rel}\left(P\right)}+\frac{\varphi_{5}}{\text{Rep}\left(P\right)}\right),\\ \varphi_{1}+\varphi_{2}+\varphi_{3}+\varphi_{4}+\varphi_{5}=1. \end{array}\right. \end{array}$$

## 3. Service Composition Method Based on BAS Optimization Algorithm

### 3.1. BAS Algorithm Analysis

We transform beetle antenna search into an optimization problem in *n*-dimensional space, using *x*_*l*_ as left antenna coordinate, *x*_*r*_ as right antenna coordinate, *x* as mass center coordinate, and *d* as distance between two antennas. Since the beetle's head orientation is arbitrary, as well as a standardized random vector can be generated from beetle's right antenna pointing to its left antenna. The standardized random vector is shown in the following formula:

(14) $$\begin{array}{c} \overset{⟶}{b}=\frac{\text{rands}\left(n,1\right)}{\left\|\text{rands}\left(n,1\right)\right\|}. \end{array}$$

The generated random vector (beetle's right antenna pointing to its left antenna) is shown as follows:

(15) $$\begin{array}{c} x_{l}-x_{r}=d*\overset{⟶}{b}. \end{array}$$

At *t* moment, if position of beetle is *x*^*t*^, the coordinates of left and right antenna are shown as follows:

(16) $$\begin{array}{c} x_{l}^{t}=x^{t}+\frac{d}{2}*\overset{⟶}{b},x_{r}^{t}=x^{t}-\frac{d}{2}*\overset{⟶}{b}. \end{array}$$

If odor function is *f* (x), the values of the left and right antennas are shown as follows:

(17) $$\begin{array}{c} f_{\text{left}}=f\left(x_{l}\right),f_{\text{right}}=f\left(x_{r}\right). \end{array}$$

At *t* − 1 moment, if *f*_left_ > *f*_right_, then the beetle moves left, and beetle position in next moment is $x^{t}=x^{t-1}+\text{step}*\overset{⟶}{b}$. If *f*_left_ < *f*_right_, then beetle moving right, and beetle position in next moment is $x^{t}=x^{t-1}-\text{step}*\overset{⟶}{b}$. According to this rule, we use the following formula to express beetle moving position in next moment:

(18) $$\begin{array}{c} x^{t}=x^{t-1}-\text{step}*\overset{⟶}{b}*\text{sign}\left(f_{\text{left}}-f_{\text{right}}\right). \end{array}$$

Considering that beetle search step size will decay with increase of time, and a single beetle easy to lose in search process, we use *n* beetles search measures to circumvent these problems. Randomly select *n* individuals with different step lengths to spatial search, and keep each beetle step length unchanged throughout search period, and step length of each beetle is determined by the size of beetle itself; then, step expression can be expressed as follows:

(19) $$\begin{array}{c} {\text{step}}_{i}={\text{Cd}}_{i},\left(i=1,2,3,\ldots ,n;C\text{ is a constant}\right). \end{array}$$

The search path, by *i*th beetle produce is expressed as *P*_*i*_, calculate and record value of QoSP_*i*_. We can calculate the probability of beetle reaching end point *ρ* and the variance *S*^2^ of evaluation function QoSP; if *ρ* > *ρ*_limit_and*S*^2^ < *S*_limit_^2^, the value of can be calculated by the following formula; otherwise, reexecute the algorithm:

(20) $$\begin{array}{c} \text{QoSP}={\text{MinQoSP}}_{1},{\text{QoSP}}_{2},\ldots ,{\text{QoSP}}_{n}\\ =\text{Min}\left(\varphi_{1}T\left(P_{i}\right)+\varphi_{2}C\left(P_{i}\right)+\frac{\varphi_{3}}{\text{Av}\left(P_{i}\right)}+\frac{\varphi_{4}}{\text{Rel}\left(P_{i}\right)}+\frac{\varphi_{5}}{\text{Rep}\left(P_{i}\right)}\right), \end{array}$$

where *i*=1,2,3,…, *n*,  *φ*_1_+*φ*_2_+*φ*_3_+*φ*_4_+*φ*_5_=1 and *P*=*P*_*i*_ is the optimal path for beetle foraging, which is the optimal service composition when it is applied in composite edge service model.

### 3.2. Edge Service Composition Algorithm

According to the above algorithm analysis, the edge service composition specific workflow is as follows:

*Step 1*. Initialize algorithm configuration and parameters; *C* is the ratio of beetle step length to distance between two antennas; then, step/*d*=*C*.
*Step 2*. Place *n* beetles with different step lengths (randomly generated) at the starting position; let *i*=0, *k*=0, *i* represents *i*th (*i*=0,1,2,3,…, *n* − 1) beetle in this cycle, and record the initial position, *k* (*k*∈(0, *n*]) represents the number of beetles reach target position successfully.
*Step 3*. *i* = *i* + 1 and *i* < *n*, go to next step; otherwise, execute step 8.
*Step 4*. If beetle is lost, return to step 3; else, go to next step.
*Step 5*. Initialize *j* = 0, the number of cycles *N* (*N* is a constant).
*Step 6*. If *j* < *N*, then QoS(*P*_*i*_)=QoSP_*i*_^*j*^, output and record QoS(*P*_*i*_^*j*^) and *P*_*i*_^*j*^, *j*++, repeat step 6; else, go to next step.
*Step 7*. Calculate QoSP_*i*_ variance *S*_*i*_^2^ as follows:
  (21) $$\begin{array}{c} S_{i}^{2}=\frac{1}{N}\left\{{\left[\text{QoS}\left(P_{i}^{0}\right)-\bar{{\text{QoSP}}_{i}}\right]}^{2}+{\left[\text{QoS}\left(P_{i}^{1}\right)-\bar{{\text{QoSP}}_{i}}\right]}^{2}+\cdots +{\left[\text{QoS}\left(P_{i}^{j}\right)-\bar{{\text{QoSP}}_{i}}\right]}^{2}\right\}\bar{{\text{QoSP}}_{i}}\\ =\frac{1}{N}\overset{N}{\underset{j=1}{\sum }}\left(\text{QoS}\left(P_{i}^{j}\right)\right). \end{array}$$
If *S*_*i*_^2^ < *S*_limit _^2^, then QoS(*P*_*i*_)=min(QoSP_*i*_^0^, QoSP_*i*_^1^,…, QoSP_*i*_^*N*^), return the value of QoSP_*i*_ and *P*_*i*_(*P*_*i*_=*P*_*i*_^*j*^) and record them, then *k*=*k*+1, return step 3; else, execute step 5.
*Step 8*. Calculate the value of QoSP and path *P* as follows:
  (22) $$\begin{array}{c} \text{QoSP}={\text{MinQoSP}}_{1},{\text{QoSP}}_{2},\ldots ,{\text{QoSP}}_{n},\\ P=P_{i}=P_{i}^{j}. \end{array}$$

Finally, return the value of QoSP and path *P*.

## 4. Experiment and Analysis

### 4.1. Simulation Experiment Environment and Solution Target

The type of experimental computer is HP880G1, ACPI ×64-based PC. Processor is Intel^®^ Core^™^ i5-4590 CPU @ 3.30 GHz. Random access memory (RAM) is 4.0 GB. System type is Windows 8 64-bit operation system. Simulation software is MATLAB-R2018b. The objective function is expressed as follows:

(23) $$\begin{array}{c} {\text{FX}}_{i}=\text{QoSP}=\text{Min}\left(\varphi_{1}T\left(P_{i}\right)+\varphi_{2}C\left(P_{i}\right)+\frac{\varphi_{3}}{\text{Av}\left(P_{i}\right)}+\frac{\varphi_{4}}{\text{Rel}\left(P_{i}\right)}+\frac{\varphi_{5}}{\text{Rep}\left(P_{i}\right)}\right). \end{array}$$

Due to *φ*_1_+*φ*_2_+*φ*_3_+*φ*_4_+*φ*_5_=1 in formula (13), here we assign the parameters as follows: *φ*_1_=0.2, *φ*_2_=0.2, *φ*_3_=0.2, *φ*_4_=0.2, and *φ*_5_=0.2.

### 4.2. Global Optimization Ability of BAS Algorithm

**Figure 3:**
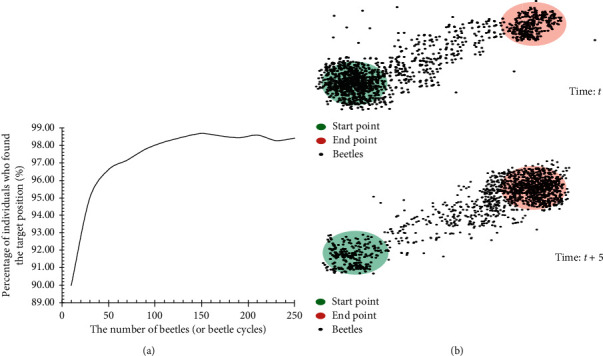
The optimization ability and state diagram of beetles. (a) The probability of reached target position. (b) State diagram of beetles at *T* and *T* + 5.

Because a single beetle with variable step length is easy to get lost in random direction in optimization process, in this paper, we use several beetles (a group of individuals with different step lengths) to perform a global optimization test, and the step size of each beetle is fixed during optimization process, and different beetles have different step lengths. In this experiment, different quantity beetles were used to test optimization ability of algorithm. The experimental results show that more than 90% of beetle can reach global optimal position successfully with a small quantity beetles. With the quantity of beetles continuous increase, the stronger the global optimization ability, as shown in Figure 3(a). The optimization state diagram of beetles at time *t* and *T* + 5 is shown in Figure 3(b).

### 4.3. The Efficiency of Edge Service Composition Method

**Figure 4:**
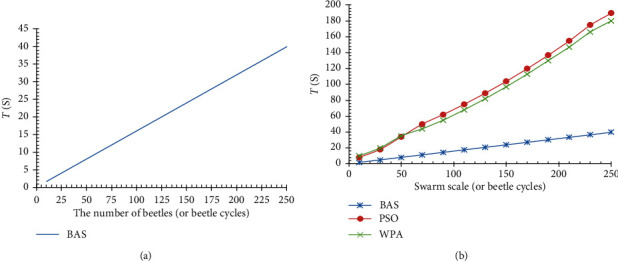
Edge service composition comparison of PSO, WPA, and BAS. (a) Relationship between time *T* and beetle cycles. (b) Comparison with PSO and WPA.

In the case of equivalent quantity edge services, the relationship between beetles quantity and time *T* is shown in Figure 4(a), and our method is significantly better than PSO, WPA, and other optimization algorithms compared with the time performance, as shown in Figure 4(b).

**Figure 5:**
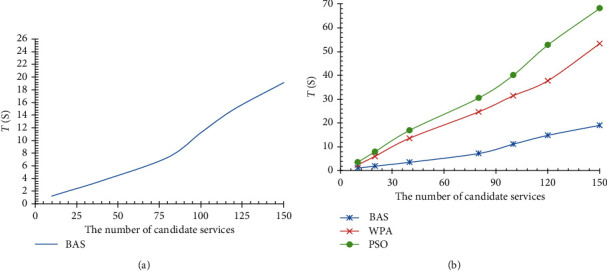
Relationship between edge service composition efficiency (time *T* and candidate service sets, comparison with other service composition methods (such as PSO and WPA)). (a) Relationship of time *T* and candidate service sets. (b) Comparison with PSO and WPA.

In the case of swarm scale (or beetle cycles), we experiment the use of different candidate edge service sets to solve the same service composition problem; the result shows that the larger we used candidate service set, the longer solution time-consuming is, and the faster the growth rate of time-consuming is. The experimental result is shown in Figure 5(a). We simulate service composition algorithms based on PSO and WPA under the same conditions, and comparison showed that the BAS edge service composition algorithm used in this paper was significantly better than PSO and WPA methods in solving service composition in time performance. The comparison results are shown in Figure 5(b).

### 4.4. Comparison between Edge Service Composition and Cloud Service Composition

This experiment builds and designs an experimental platform according to the cloud service composition model and edge service composition model (as shown in Figure 1). The platform deploys a cloud cluster and four edge servers on the edge of cloud cluster. The cloud cluster consists of a master node server and three slave node servers. Each node server or edge server is deployed in different virtual machine.

*Experiment Description*. The same service request is served by cloud service composition and edge service composition close to the service request, records and analyzes time performance from service request to complete service content.

**Figure 6:**
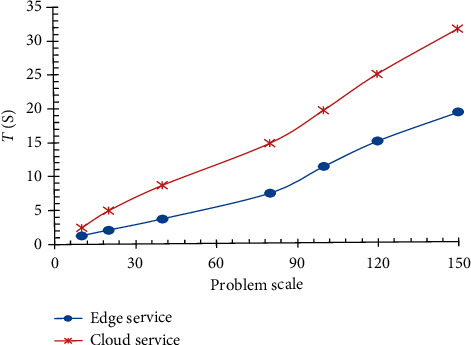
Comparison of edge service composition and cloud service composition.

The experiment results show that the closer service resource is to service request, the shorter response time to service request, the higher service efficiency, and the lower service time and cost. On the contrary, if service resource is in cloud far away from service request, the longer response time to service request, the lower service efficiency, and the service cost such as time and expense is higher relatively. As shown in Figure 6, it shows that the efficiency of edge service composition in providing services is significantly better than cloud service composition by time comparison between edge service composition and cloud service composition in providing service for service requests.

## 5. Conclusion

In this paper, an edge service composition model based on edge computing is designed, and an optimization method for edge service composition based on beetle antenna search algorithm is proposed. Compared with cloud service composition model, the edge service composition model has advantage that a large amount of computing tasks is handed by edge service equipment that is closer to service request. It has fast data processing speed, no delay in transmission, fast response, and quality of user experience and satisfaction highly. Although edge service composition method based on BAS algorithm in this paper has obvious advantages in efficiency of service composition compared with the methods based on PSO and WPA, there are still some deficiencies and needs to be improved. BAS algorithm was first proposed by Jiang and Li in 2017 [24]; there are few cases for service composition, and it has many uncertainties and possibilities. We will combine particle swarm optimization, wolf swarm optimization, or ant colony algorithm to further improve edge service composition method based on BAS from the aspect of variable step length.

## Figures and Tables

**Table 1:** Aggregate function model of QoS attributes.

Structure type				
Attributes	Sequence	Select	Parallel	Cycle
	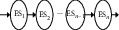	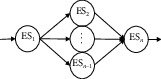	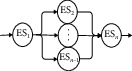	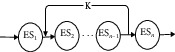
Service time (*T*)	∑_*i*=1_^*n*^*T*(ES_*i*_)	∑_*i*=1_^*n*^(*T*(ES_*i*_)*∗p*_*i*_)	Max(*T*(ES_*i*_))*i* ∈ [1, *n*]	*K∗*∑_*i*=1_^*n*^*T*(ES_*i*_) (note: *K* is the number of cycles)
Service cost (*C*)	∑_*i*=1_^*n*^*C*(ES_*i*_)	∑_*i*=1_^*n*^(*C*(ES_*i*_)*∗p*_*i*_)	∑_*i*=1_^*n*^*C*(ES_*i*_)	*K∗*∑_*i*=1_^*n*^*C*(ES_*i*_) (note: *K* is the number of cycles)
Availability (Av)	∏_*i*=1_^*n*^Av(ES_*i*_)	$\overset{n}{\underset{i=1}{\sum }}\left(Av\left(ES_{i}\right)*p_{i}\right)$	∏_*i*=1_^*n*^Av(ES_*i*_)	∏_*i*=1_^*n*^Av(ES_*i*_)
Reliability (Rel)	∏_*i*=1_^*n*^Rel(ES_*i*_)	∑_*i*=1_^*n*^(Rel(ES_*i*_)*∗p*_*i*_)	∏_*i*=1_^*n*^Rel(ES_*i*_)	∏_*i*=1_^*n*^Rel(ES_*i*_)
Reputation (Rep)	∑_*i*=1_^*n*^(Rep(ES_*i*_)/*n*)	∑_*i*=1_^*n*^(Rep(ES_*i*_)*∗p*_*i*_)	∑_*i*=1_^*n*^(Rep(ES_*i*_)/*n*)	∑_*i*=1_^*n*^(Rep(ES_*i*_)/*n*)

## Data Availability

The experimental result data used to support the findings of this study are included within the article.

## References

[1] Zeng L., Benatallah B., Dumas M., Kalagnanam J., Sheng Q. Z. Quality driven web services composition.

[2] Zeng L., Benatallah B., Ngu A. H. H., Dumas M., Kalagnanam J., Chang H. (2004). QoS-aware middleware for web services composition. *IEEE Transactions on Software Engineering*.

[3] Deng S., Huang L., Wu H., Wu Z. Constraints-driven service composition in mobile cloud computing.

[4] Ardagna D., Pernici B. Global and local QoS constraints guarantee in web service selection.

[5] Ardagna D., Pernici B. (2007). Adaptive service composition in flexible processes. *IEEE Transactions on Software Engineering*.

[6] Wang H., Zou B., Guo G., Zhang J., Yang Z. Optimal and effective web service composition with trust and user preference.

[7] Berbner R., Spahn M., Repp N., Heckmann O., Steinmetz R. Heuristics for QoS-aware web service composition.

[8] Guidara I., Jaouhari I. A., Guermouche N. Dynamic selection for service composition based on temporal and QoS constraints.

[9] Laleh T., Paquet J., Mokhov S., Yan Y. Constraint adaptation in web service composition.

[10] Alrifai M., Risse T., Nejdl W. (2012). A hybrid approach for efficient web service composition with end-to-end QoS constraints. *ACM Transactions on the Web*.

[11] Hwang S. Y., Lim E. P., Lee C. H., Chen C. H. (2009). Dynamic web service selection for reliable web service composition. *IEEE Transactions on Services Computing*.

[12] Hadad J. E., Manouvrier M., Rukoz M. (2010). TQoS: transactional and QoS-aware selection algorithm for automatic web service composition. *IEEE Transactions on Services Computing*.

[13] Chen Y., Huang J., Lin C. Partial selection: an efficient approach for QoS-aware web service composition.

[14] Chen Y., Huang J., Lin C., Hu J. (2015). A partial selection methodology for efficient QoS-aware service composition. *IEEE Transactions on Services Computing*.

[15] Deng S., Wu H., Hu D., Zhao J. L. (2016). Service selection for composition with QoS correlations. *IEEE Transactions on Services Computing*.

[16] Lin C., Chen Y., Huang J.-W. (2015). A survey on models and solutions of multi-objective optimization for QoS in services computing. *Chinese Journal of Computers*.

[17] Wang Y., Chen I. R., Cho J. H., Swami A., Chan K. (2017). Trust-based service composition and binding with multiple objective optimization in service-oriented mobile ad hoc networks. *IEEE Transactions on Services Computing*.

[18] Wagner F., Klein A., Klopper B., Ishikawa F., Honiden S. Multi-objective service composition with time- and input-dependent QoS.

[19] Cremene M., Suciu M., Pallez D., Dumitrescu D. (2015). Comparative analysis of multi-objective evolutionary algorithms for QoS-aware web service composition. *Applied Soft Computing*.

[20] Yilmaz A. E., Karagoz P. Improved genetic algorithm based approach for QoS aware web service composition.

[21] Kim M., Ko I. Y. An efficient resource allocation approach based on a genetic algorithm for composite services in IoT environments.

[22] Hossain M. S., Moniruzzaman M., Muhammad G., Ghoneim A., Alamri A. (2016). Big data-driven service composition using parallel clustered particle swarm optimization in mobile environment. *IEEE Transactions on Services Computing*.

[23] Peng S., Wang H., Yu Q. Estimation of distribution with restricted Boltzmann machine for adaptive service composition.

[24] Jiang X., Li S. (2018). BAS: beetle antennae search algorithm for optimization problems. *International Journal of Robotics and Control*.

[25] Yang J., Wen J., Jiang B., Lv Z., Sangaiah A. K. (2018). Marine depth mapping algorithm based on the edge computing in internet of things. *Journal of Parallel and Distributed Computing*.

[26] Taherizadeh S., Jones A. C., Taylor I., Zhao Z., Stankovski V. (2018). Monitoring self-adaptive applications within edge computing frameworks: a state-of-the-art review. *Journal of Systems and Software*.

[27] Ryden M., Oh K., Chandra A., Weissman J. (2017). Nebula: distributed edge cloud for data intensive computing. *IEEE Transactions on Parallel and Distributed Systems*.

[28] Wan S., Gu Z., Ni Q. (2020). Cognitive computing and wireless communications on the edge for healthcare service robots. *Computer Communications*.

[29] Chen C., Liu B., Wan S., Qiao P., Pei Q. (2020). An edge traffic flow detection scheme based on deep learning in an intelligent transportation system. *IEEE Transactions on Intelligent Transportation Systems*.

[30] Chen C., Zhang Y., Wang Z., Wan S., Pei Q. (2021). Distributed computation offloading method based on deep reinforcement learning in ICV. *Applied Soft Computing*.

